# Technological optimization and fatigue evaluation of carbon reinforced polyamide 3D printed gears

**DOI:** 10.1016/j.heliyon.2024.e34037

**Published:** 2024-07-03

**Authors:** Matija Hriberšek, Simon Kulovec, Awais Ikram, Matjaž Kern, Luka Kastelic, Franci Pušavec

**Affiliations:** a Faculty of Polymer Technology (FTPO), Ozare 19, Slovenj Gradec, 2380, Slovenia; b Podkrižnik Research Organization, Podkrižnik d.o.o., Loke 33, Ljubno Ob Savinji, 3333, Slovenia; c University of Ljubljana, Faculty of Mechanical Engineering, Aškerčeva 6, Ljubljana, 1000, Slovenia

**Keywords:** Gears, Additive manufacturing, Onyx, Polyamide, Carbon fibers, Temperature, Wear, Fatigue life

## Abstract

Nowadays, this is even more challenging while additive manufacturing technology is used for prototyping, as well as the production of gearing systems. Presented is an optimization and characterization of the additively manufactured (AM) gears made of carbon-reinforced polyamide material in correlation to conventional polymer gearings. Based on the obtained results, the fatigue life data for AM carbon-reinforced Polyamide gears is calculated and compared to traditionally produced Polyamide 66-based gears. Results show that the durability of AM-produced carbon-reinforced Polyamide gears can be compared to conventional-produced Polyamide 66-based gears. Furthermore, deduced that wear of the AM-reinforced Polyamide teeth, can be closely connected with the meshing temperature in correlation with fatigue.

## Highlights

• Topology of gears has decisive impact on durability performance.
• Onyx gear shows appropriate durabilty performance to use in prototyping concpets.
• Running-in phase has significant impact on Onyx gear deformation rate.
• During wear in phase deformation is proportionally with wear rate.

## 1 Introduction

Gears are mechanical drive elements mounted in industrial driving assemblies to transfer mechanical motion under various speeds, with suitable augmentation of feed and torque between shafts, in a particular power transmission application. In various research and industrial environments, the most used gears are spur gears, which typically offer power transmission efficiencies greater than 95 % and can effectively transfer substantial amounts of power across multiple tethered gears, compensating for negligible power loss.

The design and structure of a spur gear drastically impact its performance, and they are made either of high-strength metals, alloys, or durable plastic. Metallic spur gears are fabricated using milling, hobbing, cold drawing, hot isostatic pressing (HIP), roll forming, or die forging methods, depending on the type of material and designated application. On the other side, polymer gears are widely adopted in several industries and appliances, such as conveyor systems, speed actuators, power transmission components, pumps, engine motors, printers, mechanical transportation systems, or machine tools, due to their plausible advantages over metallic gears, such as weight reduction, noise and vibration dampening, decent tribological features (self-lubricating and low friction coefficient), corrosion resistance, and cost savings, with flexibility to produce many parts quickly (in large batch sizes) [1]. The notable drawbacks of polymer gears include creep susceptibility, reduced load-bearing capacity, poor manufacturing precision, poor thermal stability, and heat conductivity [2]. Conventionally, polymers for gears comprise of cast or injection-molded polyamides, polyesters (PE), polylactic acid (PLA+), and polyacetal (POM) with numerous filler reinforcements to strengthen the base, such as glass fibers, carbon fibers, aramid fibers (Kevlar), and other additives to enhance the lubricity, such as Polytetrafluoroethylene (PTFE), silicone oil, boron nitride, etc. A wide majority of polymer gears are fabricated by injection molding methods, while hot isostatic pressing and extrusion are also common. The interest in additive methods and 3D printing is increasing due to the wide possibility of design adjustments [1]. A key area of concern with additive manufacturing is the longer production time in comparison with injection molding, thus constraining industry-wide mass adaptation of this method. Reinforced polymer gears, which have a higher strength-to-weight ratio and thermal resistance, have shown definite improvements in vibration damping, fatigue cyclic loading, contact friction, and wear [[3], [4], [5]]. Designing gears out of polymeric materials also stresses evaluating various factors, including flexural, bending, and ultimate strength criteria, thermal expansion/shrinkage, moisture absorption, and possible UV/chemical exposure.

Medium to high-power transmission functions require polymer gears that typically operate in a dry environment without any lubrication. However, this can result in a short service life of the gear drive due to rising contact friction and subsequent thermally induced wear [6]. Tsukamoto et al. [7] identified two main failure mechanisms when testing polyamide gears, with the first one attributed to mechanical factors and the second one to thermal loading. The performance criterion and mechanical properties of polymer gears are quite susceptible to temperature variations. Concurrent estimation methods for the gear's surface temperature are based on the Hachman and Strickle approach, which assumes that gear tooth heat transfer does not fluctuate significantly with lubrication [8,9]. However, Chen et al. [10] suggested that lubrication considerably enhances the mechanical performance and fatigue life of polymer gears. The typical failure modes in polymeric gears are wear, pitch cracking, fatigue, root, and pitting, with the mechanisms largely dictated by temperature fluctuations. In all cases, correct assessment of mechanical performance and capability against failures under certain loading conditions is imperative when dimensioning gear drives during the design and production phases [11]. Letzelter et al. [12] monitored the thermal behavior of Polyamide 66 polymer gears using an infrared thermographic camera and the evolution and distribution of meshing temperature depending on time to illustrate humidity-dependent viscoelastic properties. Breeds et al. [13] comprehensively determined the meshing characteristics, gear material pairing, loading conditions, operating rotational speeds, ambient temperature, and gear geometry, all of which have a crucial impact on wear progression. Moreover, the worn flanks have different features due to the direction of rolling and frictional forces along individual gear faces, so wear limits gear lifetime at lower torques while the permissible surface temperature is at higher loading. Ratansumawonget et al. [14] interpreted the major impact of friction coefficient on the sliding losses in polymer gear pairs, resulting in a decrease in power transmission efficiency.

Senthilvelan et al. [15] conducted tests on Polyamide gears reinforced with 0–20 % short carbon fibers (SCF) and found that the higher elastic modulus and strength of composites led to superior fatigue life, while the orientation of fibers had the main impact on tooth wear. Coleman et al. [16] noted that carbon nanotube (CNT) reinforcement can dramatically increase Young's modulus, tensile strength, and toughness of polymer matrix composites (PMC). Tavčar et al. [17] performed accelerated lifetime testing on (carbon, glass) reinforced polymer gears to identify factors contributing to the durability of reinforced polymer gears linked to their tribological and thermal effects within the service range. They concluded that unreinforced polymer gears are better for higher loading cycles, whereas reinforced gears have higher stress sustainability. Singh et al. [18] correlated the thermal performance and wear of Acrylonitrile Butadiene Styrene (ABS), High-density Polyethylene (HDPE), and Polyoxymethylene (POM) based gears at different torque levels (0.8–2.0 Nm) and rotational speeds (600–1200 rpm), and suggested that ABS gears fail due to extreme tooth wear, HDPE gears crack at the root segment of the tooth, while POM gears have about two-fold lower specific wear rate than the ABS parts. Li et al. [19] investigated the wear performance of dissimilar polymer gears engaged in contact and found that the geometric modification of gear flanks by tip relief can lead to a significant reduction in wear. Pogačnik et al. [20] stated that friction conditions depend on both represented materials in the gear pair and not individually for each material. Kunishima et al. [21] studied the tribological properties between PA66 composites (carbon and glass) and steel parts in contact and suggested that the SCF-reinforced PA66 experienced reduced friction and wear in contrast to both the PA66 polymer and short glass fiber (SGF) reinforced composite specimen. Lin et al. [22] modeled the dynamic contact load versus the tooth profiles of Polyamide 66 and POM-based gear pairs and realized that the cumulative sliding wear causes significant alteration of tooth profiles, which considerably affects the contact load. Senthilvelan et al. [23] characterized the damping behavior of unreinforced Polyamide 66 and 20 % carbon fiber reinforced spur gears and found that the addition of fibers to the base polymer reduces the damping capability of the composite, which in turn affects the hysteresis heating as well as the ability to absorb vibration during service. Gavali et al. [24] demonstrated direct enhancement in the tensile and bending strength, micro-hardness, elastic modulus, and glass transition temperature in PLA reinforced with 15 wt % CF fabricated by Fused Deposition Modeling (FDM). Yu et al. [25] evaluated the concentric continuous carbon fiber (CCF) infill configuration and found that it displayed superior flexural strength and energy absorption capability than those in the isotropic carbon fiber infill pattern and that the addition of CF rings enhanced the flexural strengths by 40 %.

Mao et al. [26] concluded from their study that the wear rate of polymer gears is independent of the manufacturing method utilized (e.g., injection molding, cutting, etc.). Citing the benefits of 3D printed spur gears, Rohit et al. [23] demonstrated the optimization of modeling parameters with CATIA V5 R21 and ANSYS suites prior to FDM printing on ABS and Poly Lactic Acid (PLA). Additive manufacturing methods promise the fabrication of polymer matrix composites with desired mechanical properties and complex shapes. Kotkar et al. [27] simulated the mechanical performance of Fused Deposition Modeling (FDM) 3D-printed polymeric spur gears and found that inclined Polyamide 66 was the most appropriate material for power transmission applications. Ghebretinsae et al. [28] utilized a Markforged® Mark II 3D printer to fabricate Onyx (a thermoset polymer-CF composite) samples with ultimate tensile strength (UTS) of 560 MPa, tensile modulus of 25 GPa, flexural strength of 271 MPa, and flexural modulus of 16 GPa, suggesting extensive feasibility of this material for high-performance polymer gears. Zhang et al. [29] assessed the performance of various 3D-printed gears including Polyamide 618, Polyamide 645, and Alloy 910 filaments, as well as Onyx and Markforged polyamide. Based on their wear rate test results on custom-built test benches, Polyamide 618-based 3D-printed gears demonstrated better wear performance compared to injection-molded polyamide 66 gears at low to medium torque range. Anand et al. performed studies of the bending strength of 3D printed composite gears to determine the maximum tensile bending stress developed in gear using single tooth specimens [30]. Yilmaz et al. [31] observed the mechanical characteristics of hybrid gears composed of high-strength steel and carbon fiber-reinforced plastic. The author studied the effect of rack tip radius, drive side pressure angle, and rim thickness on the stress of hybrid gears using Finite Element Modeling. They found out that increasing of rim thickness has a positive effect pon the stress and static transmission error, while drive side pressure angle is effective on the dynamic factor at most. Chidambaram et al. [32] performed characterization of natural composite materials which consists of stir-formed Araldite LY 109 epoxy resin and Hardner – Aradur with coir-reinforced. The authors studied the mentioned natural composite to integrate it into a helical gear due to its material benefits such as good hardness, impact, and wear resistance. They compared measured quantities to currently used materials for helical gears which are Araldite LY 109 epoxy resin and Hardner. They found out that the proposed natural composite has better abrasion and tensile strength compared to the currently used material. Černe et al. [33] conducted a comprehensive investigation of the wear properties of carbon fiber polymer gears manufactured by an autoclave curing process. The composite gears were mated with 42CrMo4 steel pinion and subjected to nominal bending stresses between 60 and 150 MPa. They used an infrared thermal camera for spot temperature detection, while wear rates were measured at regular intervals. The finite element method was used to research the in-mesh contact and root stress behavior of gears under various loads and running times. The results of the study indicate that although wear, even at low levels, significantly affects transmission error, the relationship between these two parameters is typically not linear. Bergant et al. [34] performed a study where fatigue and wear characteristics of carbon-fiber reinforced gears cured by autoclave were performed. The proposed gears produced by a composite were mated with 42CrMo4 steel gears ranging from 60 to 150 MPa. A finite element method was used to examine the in-mesh contact and root stress behavior of both new and worn gears under various loads and a specific running time. The results have shown that new composite gears exhibited superior performance compared to conventional plastic and composite short-fibrous gears. Shil'ko and Starzhinski [35] developed a method to determine wear resistance of materials reinforced by particles or randomly oriented fibers. The method is based on the fatigue theory applied for the estimation fatigue life of the reinforced polymer gears. The results showed that carbon fiber-reinforced plastics are superior to glass fiber-reinforced ones owing more favorable combination of frictional and mechanical properties. The numerical results confirm experimental findings. Chernets et al. [36] performed tribotesting of the polyamide composite dispersely filled with glass and carbon fibers. The results showed four times better wear resistance of carbon-reinforced polyamide compared to glass-reinforced polyamide. The calculated lifetime of carbon-reinforced polyamide is 8.3 longer compared to glass-reinforced polyamide gear. Lingesh et al. [37] studied the mechanical properties of 10–20 % carbon-reinforced PA66 composites and observed an increase in tensile and flexural strengths with a reduction in elongation. Similar mechanical properties boost for hybrid composites was also confirmed by Chavan et al. [38] with short glass (SGF) and carbon fiber (SCF) in the PP/PA66 matrix. Barnik et al. [39] characterized the mechanical properties of Onyx-based composites with different densities and shapes of fill, suggesting that the mechanical integrity is poorer for an odd number of layers than for an even number of layers, and the gear specimen broke along the plane in which a smaller number of layers were deposited. Regarding the tensile strengths, the specimens with higher infill densities yielded better ultimate tensile loading (UTS). Mulholland et al. [40] verified the microstructure-property relationship after fused filament fabrication (FFF) of carbon fiber-filled polyamide parts, suggesting that the thermal conductivity (anisotropic heat exchange) increased by more than three-fold along the fiber orientation, at a loading capacity of merely 12 % vol, and only partially in the other directions.

Therefore, this study aims to evaluate the effectiveness of additively manufactured (AM) spur gears made with high-performance Onyx polyamide resin, which boasts superior mechanical properties due to its carbon reinforcement. To determine the benefits of these gears that are AM over traditional machine-cut polymer gears, fatigue and wear were assessed. The study thus utilized Fused Filament Fabrication (FFF) additive manufacturing techniques. Additionally, the study aimed to compare the performance of additively manufactured carbon reinforced Polyamide (Onyx) gears with Polyamide 66 polymer gears reinforced with 20 % carbon fibers, which were machined to the same geometric design. The objective was to evaluate the suitability of AM gears to be used in power transmission applications during the prototyping phase of mechanical part development. Previous research has reported on the wear characterization of FFF, or 3D printed composites, as well as polymer gears, through simulation and experimental sequences. However, the suitability of Onyx for fabricating serviceable spur gears has yet to make a distinctive impact in the literature. To the best of our knowledge, there is a dearth of scientific reports on wear and fatigue assessment for 3D-printed Onyx-based spur gears. Thus, this study aims to fill the gap in the total assessment of in-service mechanical properties, including flexural, wear, and fatigue, of SCF spur gears, along with the 3D printing parameters.

## 2 Experimental work

### 2.1 Material properties

The material used in the additive manufacturing process was Onyx (carbon fiber reinforced polyamide), which is a composite material consisting of engineering polyamide (PA66) and micro-carbon fibers. To better understand the performance of Onyx, equal test procedures were conducted for machine-cut Polyamide 66 reinforced with 20 % carbon fibers (PA66 CF20). Table 1 below shows the basic mechanical and physical properties of the materials used in this research [28].

**Table 1:** Material properties of the polyamide 66-based materials obtained at normal conditions cited from the lietrature [28,41].

Material properties (Mean Value)	Onyx	Machine cut PA66 CF20
Tensile Modulus [MPa]	1400	12000
Ultimate Tensile Strength [MPa]	36	190
Flexural Strength [MPa]	81	300
Flexural Modulus [MPa]	3600	11000
Poisson ratio [−]	0.43	0.4
Heat Deflection Temperature [°C]	145	145
Density [g/cm^3^]	1.18	1.23

Van der Klift et al. [41] analyzed to determine the fiber volume fraction of Markforged® carbon fiber filament by using the JIK K7075 standard, which involves evaporating the matrix. They found that the fiber volume fraction was 34.5 %, while the rest (65.5 %) comprised of other coating and adhesive polymers. This estimation was utilized to calculate the structural properties of the CF filament provided by Markforged® in the present study. Table 1 displays the mechanical and physical properties of Onyx and Polyamide 66. Onyx exhibits superior flexural strength and heat deflection rating over 145 °C compared to Polyamide 66 while maintaining a similar density of 1.1 g/cm^3^. To estimate the Poison's ratio of Onyx, five samples were tested, and it was approximated to be 0.43 [28,41].

### 2.2 Tested gear geometry

**Fig. 1:**
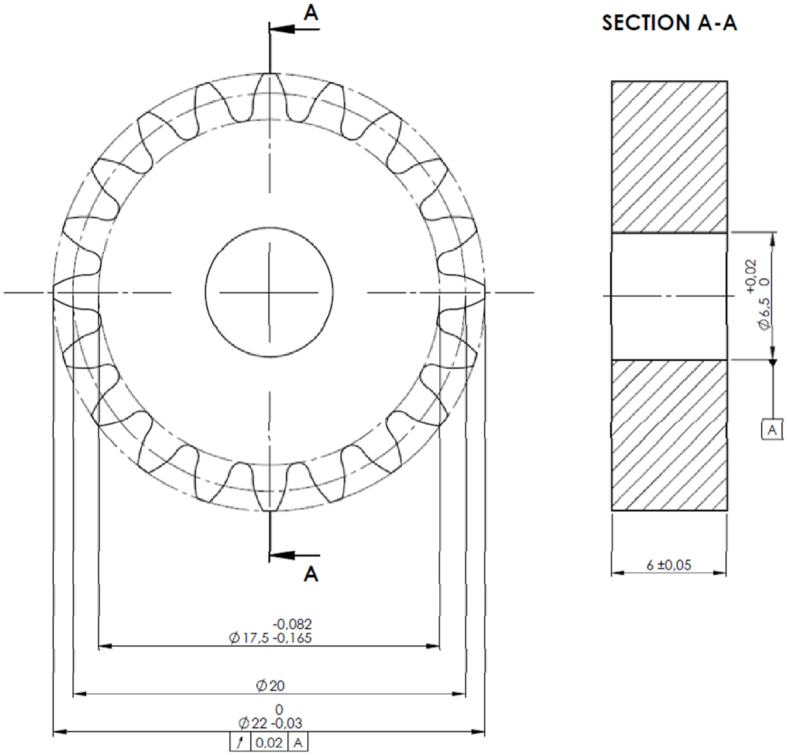
Geometric top-down CAD model of the spur gear.

In all the experiments carried out, a steel driving gear was meshed with a selected driven polymer composite gear, made of Onyx or PA66 CF20 in a steel/polymer composite arrangement. All tested gears had the same geometry, which was measured according to ISO standard 1328-1. The spur gears were designed in a CAD suite according to the ISO 53.2:1997 standard profile, following the model of Podkrižnik d.o.o, Slovenia. Table 2 presents the basic geometry parameters of the additively manufactured gear, which were further tested for surface integrity, wear, and fatigue. Onyx gears were formed using a triangular filling pattern in the FFF process. A 2D drawing of the tested gear geometry is shown in Fig. 1.

**Table 2:** Tested gear geometry.

Geometric Feature	Geometry of the tested gears Steel, Onyx, PA66 CF20 test gears
Gear Teeth number, *z* [−]	20
Gear width, *b* [mm]	6
Modulus, *m* [mm]	1
Pressure angle at normal section, *α* [°]	20
Tip diameter, *d*_a_ [mm]	22
Reference diameter, *d* [mm]	20
Root diameter, *d*_f_ [mm]	17.5

**Table 3:** Printing parameters of the test gears.

3D Printing Settings		Gear 1	Gear 2
Filament Thickness [mm]		1	1
Infill Pattern		Triangles	Triangles
Infill Density [%]		100	100
Flow Rate [%]		100	105
Initial Print Temperature [°C]		130	130
Print Temperature [°C]		220	220
Print Temperature Layer 0 [°C]		240	240
Print Speed [mm/s]		40	40
Raster Speed [mm/s]		120	120
Support Infill Rate [mm/s]		15	15
Top-Bottom Thickness [mm]		2	2
Top Thickness [mm]		2.5	2.5
Wall Line Count		6	6
Zig-Zag Infill		True	True
3D Printing Results:	Top Side		
	Bottom Side

### 2.3 The 3D printing process

The spur gears were additively fabricated using Markforged Onyx One printer with Eiger® Accelerator Software and Markforged Onyx filament. The FFF process involved building components by forming consecutive layers on top of a semi-liquid thermoplastic material above its glass transition temperature (*T*g) to reduce deformation due to linear shrinkage of the polymer. The FFF printing was conducted at angles of 0° and 90° relative to the surface and nozzle feed, respectively, with a printing speed of 40 mm/s and a rastering rate of 120 mm/s. No additional support material was used during the fabrication of the Onyx-based spur gears, and no finishing processes were applied to the print surfaces after production. The printer's build space dimensions were 320 × 132 × 154 mm, allowing for a height variation (layer thickness) of 100–200 mm. The Onyx composite gears were printed at a stable temperature of 280 °C, with the build table heated to 100 °C. For a 1.75 mm thick Onyx filament, a nozzle diameter of 0.4 mm was operated to produce a layer, using a solid material filling pattern during the six passes and two flow rate profiles presented in Table 3. The accuracy of the produced gears was estimated to be lower than ±0.05 mm. In the results section, Gear 1 represents the non-optimized gear fabricated with 3D printing parameters, while Gear 2 represents the optimized gear with modified processing parameters.

When 3D printing gears, several parameters were adjusted during optimization (Table 3). Based on the optimization process, it has been found that flow rate has the most significant impact on the quality of the 3D print. The filling (infill density) was always set to 100 %, as this way we should achieve the best (100 %) filling of our printed gear. In practice, due to the different flow of the filament and the printing pattern, small voids also appear in the product, which can significantly affect the mechanical properties of the product. By increasing the flow rate of the filament, it was achieved a greater flow of filament, which made it possible to fill these voids and make the final product stronger. It is also necessary to pay attention to the dimensional adequacy of the final product. By increasing the filament flow, we also have a significant impact on the size of the final product. By setting the filament current to 105 %, it was achieved the best compromise between the quality of the product and its dimensional accuracy.

Parallely, the fabrication process of the test specimens was performed with the aim of characterizing the standard mechanical properties of the material Onyx. The specimens were produced using the same technological guidelines presented in Table 3 for Gear 2.

To characterize the mechanical properties of Onyx material, standardized ISO mechanical tests were carried out. Tensile tests were performed by the ISO 527-1 standard [42] on the Shimadzu AG - X plus testing machine, equipped with a 10 kN load cell. The specimens were fabricated according to ISO 527-2 (type BA) [43]. The tensile tests were conducted at a crosshead velocity of 1 mm/min up to an elongation of 0.25 % and then at 50 mm/min until failure. The strain was measured at the center of the span of each specimen using a Shimadzu TRViewX optical extensometer. The measuring length was 20 mm and the clamping distance was 50 mm. The bending tests were performed by the ISO 178 [44] standard on a Shimadzu AG - X plus universal machine with a 10 kN load cell. The manufactured test specimens had standardized dimensions (80 mm × 10 mm x 4 mm). The distance between the supports was 64 mm. The tests were carried out at a crosshead speed of 2 mm/min. The Charpy impact strength measurements were performed by ISO 179 [45] using a LIYI LY-XJJD5 pendulum impact tester using notched test specimens. The dimensions of the test specimen were 80 mm × 10 mm x 4 mm. The distance between the supports was 60 mm. The pendulum has an impact velocity of 2.9 m/s with an energy of 2 J for notched specimens. The TrapeziumX software (version 1.3.1) was used to process and evaluate the results [46]. Table 4 presents the measured standard mechanical properties of the fabricated specimens using Onyx material.

**Table 4:** Measured mechanical properties of the Onyx test specimens at standard laboratory conditions.

Measured quantity	ISO standard	Average	Standard deviation
Tensile break stress [MPa]	527–1:2019	35.01	1.83
Flexural strength [MPa]	ISO 178:2019	37.71	0.86
Notched Impact strength [kJ/m^2^]	ISO 179–1:2023	27.78	4.45

**Fig. 2:**
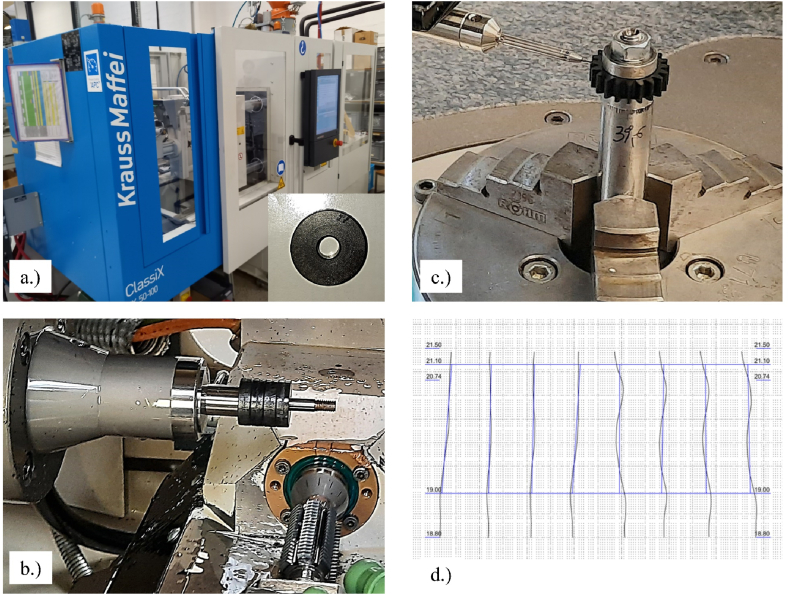
Traditional manufacturing process of testing gears from Polyamide 66 with 20 % carbon fibers: a.) Injection molding process, b.) machine cut (hobbing) process c.) gear geometry inspection with 3D coordinate measuring device, and d.) measuring report of the teeth profile deviation according to ISO 1328-1.

To compare the power transfer efficiency, thermal response, wear rate, and fatigue sustainability of the AM Onyx gears have been tested and compared conventionally manufactured Polyamide 66 reinforced carbon gears. This process involved injection molding of semi-round parts, followed by machine cutting (hobbing) of an involute tooth profile. Before processing the polymer, the material was dried at a specific temperature to achieve the recommended humidity level from the supplier. The cylindrical parts were injection molded using a Krauss Maffei ClassiX CX 50–100 machine, and the involute gear profile was machined using a hob cutting tool on a CNC machine (Koepfer 200) through a dry machining process. Fig. 2a illustrates the injection molding process of semi-round parts, while Fig. 2b shows the machine cutting (hobbing) process. Fig 2c depicts the 3D inspection process of main gear parameters, conducted by ISO 1328-1.

The steel gears, were produced of material 42CrMo4 + QT (quenched and tempered) and were manufactured from a steel bar through a turning process to machine round steel bases. An involute cutting tool was then used to generate the teeth profile. To improve the surface roughness of the steel gear flanks and to remove burrs from the gear edges, a vibratory superfinishing process was conducted. The important gear parameters were geometrically inspected using the 3D coordinate measuring device Wenzel LH54 (Fig. 2d) to ensure the appropriate class of all test gears (steel gears, Onyx gears, PA66 CF20 gears). Each gear's quality was evaluated according to the ISO standard 1328-1. The following gear parameters were inspected on all gears: profile slope, flank line, pitch deviation *f*_p_, cumulative pitch deviation *F*_p_, and runout *F*_r_. The overall quality of the steel gear was 6, while it was 11 for the Onyx gear and 8 for the PA66 CF20 gear.

### 2.4 Testing setup

**Fig. 3:**
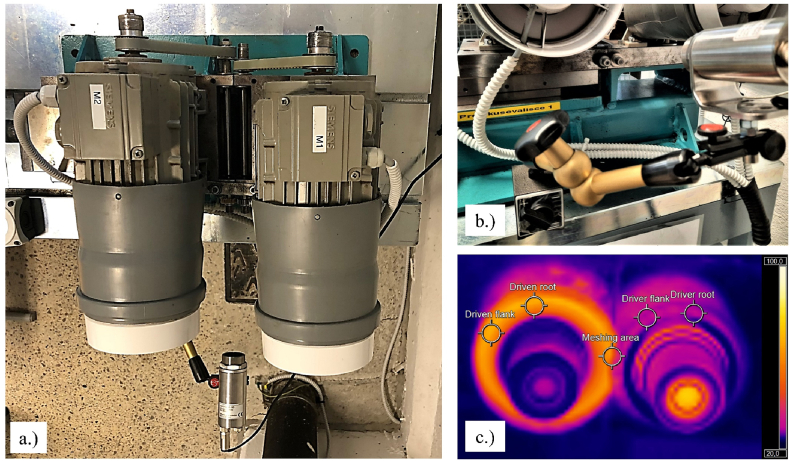
Testbench for durability testing of Onyx gears: a.) top view of the test rig, b.) side view of the test rig, and c.) Infrared image of the test gear pair (left Onyx gear and right steel gear).

The durability tests were conducted on both the non-optimized (preliminary prototypes) and optimized involute 3D printed Onyx gears using a test rig, as depicted in Fig. 3a and Fig. 3b The test rig comprises two 3-phase (4-pole) asynchronous squirrel cage electro-motors, each with a frequency of 50 Hz and power of 0.37 kW, connected to two 1-phase frequency converters (0.4 kW). The frequency converters regulate the speed of the driving gear 1 and the driven gear 2 motors. To facilitate efficient heat dissipation from the motors, two fans were mounted on the test rig. The center distance of the gear pair, was precisely set using a linear rail with a precision of 0.001 mm. The rotational motion was transmitted from the asynchronous motors to the driving and driven shafts, to which the respective gears were mounted, using a belt with a selected ratio on the pulley. The rotational speeds of each motor were controlled using the frequency converter, with motor 1 acting as the driving source and motor 2 as the braking source. The applied load level on the shafts/gears was determined as the difference between the excitation frequency of motors. The torque applied to the gear pair was calibrated experimentally by measuring the torsional deformations of the shafts using a laser beam at different excitation frequencies of the motors. The test rig was set to stop when the working electric current decreased by 5 %, indicating a root fracture of one tooth on the test gear in most cases. The center distance between the two gears was determined by considering the thermal expansion of the polymer material, with a clearance of 0.3 mm based on analytical mechanical calculations and experience.

The average spot temperature in the gear meshing contact was measured using an infrared thermo-camera (Fig. 3c The measuring area was 2 × 2 mm, with the measuring device connected to a computer to visualize temperature distribution on the gears, synchronized with other measurements. The measuring device had a frame rate of 50 Hz and a display frame rate of 20 Hz. The emissivity of the polymer material was calibrated and set to 0.95 in the thermo-camera acquisition software, with a deviation in temperature measurement of up to 1 %. Table 5 presents the technical specifications of the test bench for durability testing of polymer gears.

**Table 5:** Technical specification of the test rig.

Component	Quantity	Characteristics
Frequency-controlled 3-phase (4 poles) ASM motors (driving/driven)	2	*P* = 0.37 kW, *n* = 2000 RPM
1-phase frequency converter	2	*U* = 200 V, *P* = 0.4 kW
Fans (removing heat from motors)	2	*U* = 230 V, *f* = 50 Hz, *P* = 15 W, *Φ*_v_ = 0.027 m^3^/s
Infrared thermo-camera	1	Spot measuring area: 2 × 2 mm, Frame rate 50 Hz, display frame rate 20 Hz

To ensure statistical validity and repeatability of the process, gear pairs were tested under at least three different loading conditions and with three repetitions per experiment at each loading point. The gears were operated at a fixed rotational speed of 1400 rpm, and the tests were conducted under ambient conditions to assess the room temperature behavior of the 3D-printed Onyx gears.

### 2.5 Optical surface characterization

**Fig. 4:**
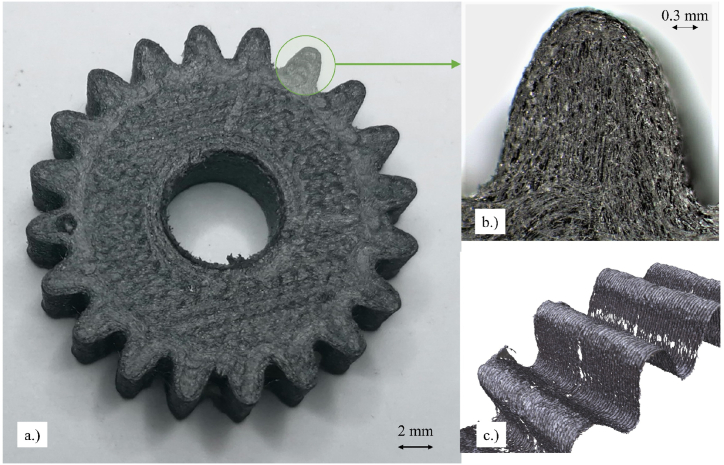
a.) optical microscopic image of 3D printed Onyx gear, b.) magnified segment of the tooth, and c.) surface topography scan of gear teeth.

The Alicona InfiniteFocusSL measurement system was utilized for the topographical characterization of the 3D printed Onyx gears. The gears were secured in both flat and tilted positions within the specimen holder and illuminated with top-down optical lighting. This microscopy system takes advantage of the optical shallow depth of field, and the topographic data was collected by vertically scanning (via piezoelectric positioning system along the z-axis) and shifting the focal lengths on randomly selected length profiles in the x-axis direction. A 3D scanning process of the worn gear profiles was performed using a precise optical microscope with a ten times magnification. The measuring area ranged from 2 to 10 mm depending on the tooth size. The measuring system resolution was set to 100 nm in the vertical direction and 4 μm in the lateral direction. The contrast of a monitored image was set to 1, and the exposure time was 1.4 ms. The topography of the gear form is presented in Fig. 4a for the optimal fabrication (Gear 2). Fig. 4b provides a closer view of the individual teeth profile and the structural micro carbon fiber-rich composition, while Fig. 4c shows the 3D scan of the printed layers, which was also generated by the Alicona system.

To normalize the wear rate of the Onyx and Polyamide 66 carbon reinforced gears, a calculation of wear coefficients according to VDI guidelines was conducted [11]. One of the most widely used theories is the standardization of wear when dry running according to the VDI2736 guideline. According to the VDI 2736 guideline, the permissible linear wear of *W*_zul_ is characterized by Eq. (1):

(1) $$W_{zul}=\left(0.1\ldots 0.2\right)∙m$$

Testing steel/polymer gear pair, tooth root failure is one of the most frequently observed failure mechanisms. During continuous gear operation, wear of teeth flanks appears, which contributes to material loss on the teeth flanks, causing changes in load carrying capacity of the gear. It is desirable to compensate for reduced load-carrying properties of the gears, with a safety factor, when calculating permissible root stress. The average linear wear *W*_m_ is characterized by Eq. (2):

(2) $$W_{m}=\frac{T_{d}∙2∙\pi ∙N_{L}∙H_{V}∙k_{W}}{b_{w}∙z∙l_{Fl}}$$

where *T*_d_ is torque, *N*_L_ number of load cycles for tested gear, *H*_v_ degree of tooth loss, *k*_W_ wear coefficient, *b*_w_ the width of the tooth, and *l*_Fl_ the profile length of the active tooth flank. The degree of tooth loss *H*_V_ is defined with Eq. (3):

(3) $$H_{V}=\frac{\pi ∙\left(u+1\right)}{z_{2}∙\cos\;\beta_{b}}∙\left(1-\varepsilon_{1}-\varepsilon_{2}+\varepsilon_{1}^{2}+\varepsilon_{2}^{2}\right)$$

where *u* = 1 is the gear ratio, *z*_2_ = 20 stands for the number of teeth, and *β*_b_ = 0 [°] is the helix angle at the base circle. The partial contact ratios *ε*_1_ of the driver and *ε*_2_ of the driven gear can be calculated with Eq. (4):

(4) $$\varepsilon_{i}=\frac{z_{i}}{2∙\pi }∙\left(\sqrt{{\left(\frac{d_{ai}}{d_{bi}}\right)}^{2}-1}-\tan\;\alpha_{wt}\right)$$

where *z*_1,2_ = 20 are numbers of teeth, *d*_a1,2_ = 22.000 mm addendum circle diameters, *d*_b1,2_ = 18.794 mm base circle diameters, and *α*_wt_ = 20° operating pressure angle in the transverse section. The profile line length of the active tooth flank *l*_Fl_ is calculated from Eq. (5) [4]:

(5) $$l_{Fl}=\frac{1}{d_{b}}∙\left({\left(\frac{d_{Na}}{2}\right)}^{2}-{\left(\frac{d_{Nf}}{2}\right)}^{2}\right)$$

where *d*_Na_ = 21.979 mm reflects the active tip diameter, and *d*_Nf_ = 18.927 mm the active root diameter [11].

## 3 Results and discussion

### 3.1 Durability testing

Durability testing was conducted to assess the performance of polymer composite gears when paired with a steel driving gear. The gear pairs tested included steel/Onyx (non-optimized as Gear 1), steel/Onyx (optimized as Gear 2), and steel/PA66 CF20 (machine cut). The load levels used for testing were 1.5 Nm, 1.3 Nm, and 1.1 Nm, with three repetitions per experiment at each load level to ensure repeatability and statistical validation. The meshing rotational speed was 1400 min^−1^ per each experiments under the prescribed load. These load levels were determined based on preliminary testing using various types of Polyacetal and Polyamide-based gears. The objective was to describe the relevant lifetime of each polymer composite material, which ranges from 100,000 to 10,000,000 load cycles. This lifetime range is critical for determining finite fatigue strength, which is essential for improving structural calculations for loaded machine parts.

#### 3.1.1 Gear meshing temperature characterization

**Fig. 5:**
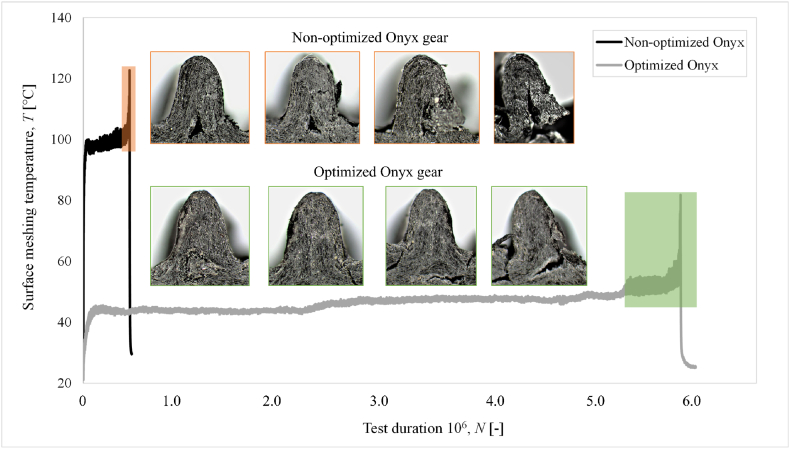
Surface meshing temperature-time diagram for evaluating the non-optimized and optimized printed Onyx gear under 1.1 Nm torque.

The preliminary durability tests were conducted on non-optimized 3D-printed Onyx gears (Gear 1) to validate the test rig and its parametric features. The prototype Onyx gears were tested at a torque of 1.1 Nm, and relevant data were obtained for a sufficiently long duration at this load level. The results of the temperature-load cycle distribution for the non-optimized (Gear 1) and the optimized (Gear 2) Onyx gear, under 1.1 Nm torque are presented in Fig. 5. Additionally, in Fig. 5, there are presented microscopic images of the crack progression for each gear that progressively occurred in the certain gear area.

The investigation revealed that the **non-optimized gear** had an unfilled part in the root area due to the non-optimized additive manufacturing process which resulted in tooth root fracture. This failure of non-optimized gear occurred at 445.632 load cycles with an associated surface meshing temperature of 122.7 °C. The repetition of these experiments for the non-optimized gear under the same loading conditions returned values of 506,369 and 525,117 load cycles before the failure of gear teeth, while the stabilized meshing temperature in the wear-in phase was identical. Microscopic analysis of the non-optimized gear revealed cracks on the surface of the pitch point and root diameter of the gear. This cracking is a result of the Hertzian contact pressure at the pitch point, where the bending loads cause tensile stresses in the root area, leading to premature rupture at lower fatigue cycles.

In contrast, in the tooth root area of **the optimized Onyx gear** crack occurs. It was evident from the non-optimized gear that the unfilled part under the root area considerably reduced the teeth's bending strength what drastically affects on the rapid crack progression.

The optimized Onyx gear has a significantly lower surface meshing temperature (45 °C) in the wear-in phase of running which has a decisive impact on the 10 times longer durability of optimized gear.

Considering three repetitions of the durability experiments for optimized Onyx gear, the average calculated lifespan of the optimized gear under load 1.1 Nm was 6.27 million load cycles with a standard deviation of 1.36 million load cycles.

**Fig. 6:**
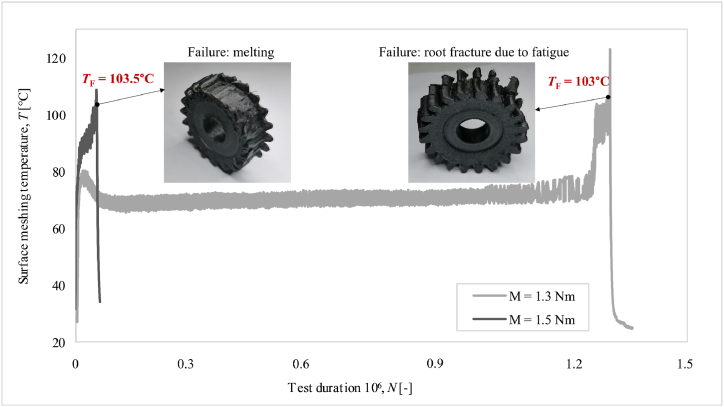
Surface meshing temperature-time diagram for optimized Onyx gears under higher loading conditions: 1.3 and 1.5 Nm torques.

Fig. 6 shows the examination of the surface meshing temperature depending on the load cycles of the optimized Onyx gears under 1.3 Nm and 1.5 Nm torquing. The calculated average of load cycles for experiments under 1.3 Nm was 2.05 million loading cycles with a standard deviation of 0.53 million load cycles, while for experiments under 1.5 Nm torque, only 0.06 million load cycles with a standard deviation of 0.01 million load cycles were sustainable. This higher torque resulted in a significant reduction in the lifespan of the Onyx gears, indicating that the material may not be suitable for applications under high loads.

In Fig. 6, it is evident that there is a constant increase in temperature throughout the test for the highest torquing conditions of 1.5 Nm. This implies a relatively rapid failure of the Onyx gear which was at 0.06 million cycles. However, it cannot be assumed to be a fatigue failure, since the excessively high temperatures caused widespread melting of the polymer matrix due to the overloading conditions (as seen in the left inset image of Fig. 6) with the corresponding temperature of the melting failure of the gear *T*_F_ = 103.5 °C. The failure temperature presented in Fig. 6 is approximately in the same range between 100 and 105 °C. According to the presented material properties in Table 1, the experimentally determined deflection temperature is lower than the cited temperature from the literature which could be the result of the fact that the gears were exposed to cyclic operation/fatigue, which over time weakened the material molecules in the structure and therefore more quickly destroyed the cohesion of the matrix and fibers and caused premature failure. For the experiment under 1.3 Nm is typical that fracture occurs in the root in the final stage of testing, at which point temperature spikes characterized as a temperature fracture *T*_F_ = 103 °C as well (as seen in the right inset image of Fig. 6), suggesting excessive wear and fatigue of the matrix material.

In the initial part of the experiments for torque 1.1 Nm (Fig. 5) and 1.3 Nm (Fig. 6), a running-in phase was identified where a transient course of temperature was detected. Here, the surface temperature increased due to teeth flank adaptation of contact, which consequently exhibits deviation of teeth profile deviation from the designed profile during additive manufacturing. This phase lasts until the teeth flanks align with each other and thus reach the wear-in phase, which can be designated with an extended period at a constant meshing temperature.

#### 3.1.2 Wear and fatigue characterization

**Fig. 7:**
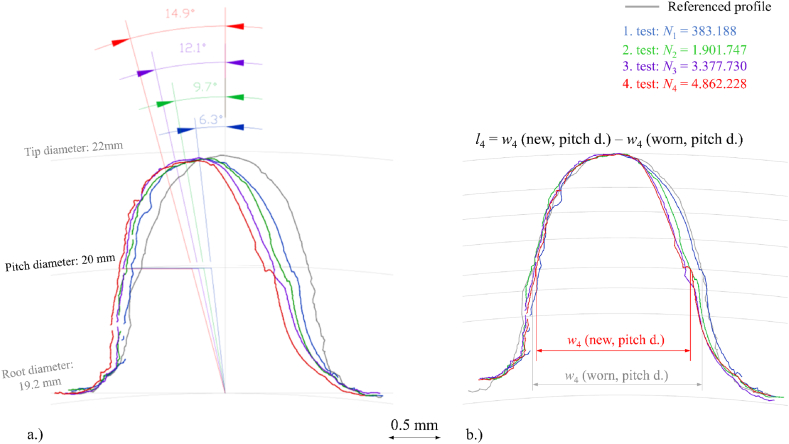
Onyx worn profiles, a.) value of the angle of rotation at pitch diameter for Onyx gear and b.) calculation of the linear wear between referenced and worn profiles at certain tooth diameters.

To assess the wear on the tooth flank of the Onyx gear, lifetime durability testing of Onyx gear pairs was conducted under a 1.1 Nm load level and was stopped at certain time intervals. The torque level of 1.1 Nm was determined based on the necessity of achieving a sufficiently long lifespan for the gear, which would cause pronounced wear on the tooth flank. After each test stop, a 3D topographical scan of the same tooth was conducted to evaluate the 3D and 2D planar wear distribution of the Onyx gear tooth. Fig. 7 shows the 2D profiles of the observed worn tooth of the Onyx gear for each measuring iteration (N1 – N4), including the referenced unworn profile. In Fig. 7a, the value of the angle of rotation at the pitch point is given for each worn profile, because of the plastic deformation due to bending loads during the meshing process. The worn profiles are aligned in the root area, where the undeformed gear region is located. The angle of rotation was measured at the pitch point where the maximal Hertzian pressure on a single tooth during the meshing process is theoretically expected.

Fig. 7b shows aligned worn profiles at the tip diameter point to illustrate the linear wear calculation procedure between the referenced and worn profiles at specific diameters. The average linear wear was measured at seven different gear diameters, and the results are plotted in Fig. 7b. The purpose of evaluating wear at the first-time sequence was to analyze the wear behavior after the running-in phase, where Onyx's teeth flanks adapt to steel's gear flanks. . Further measurements were taken at approximately equal time intervals to investigate the tooth flank wear progression of the Onyx gear.

**Fig. 8:**
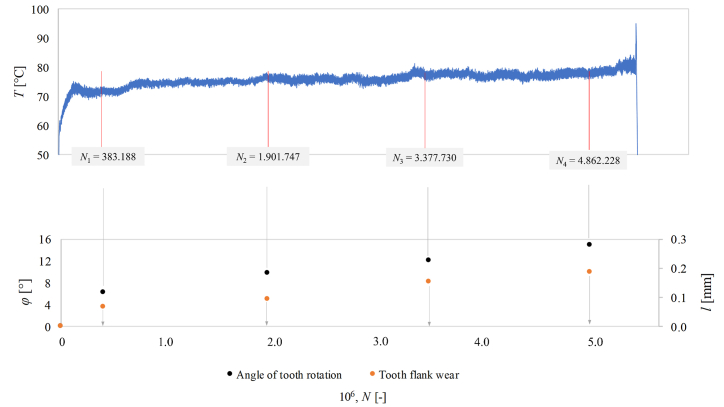
Meshing temperature cycle diagram and associated deformations with average calculated wear at prescribed measuring iterations.

Fig. 8 schematically depicts the meshing temperature-load cycle diagram for testing the steel/Onyx gear pair at the load level of 1.1 Nm with four-time sequences/iterations (*N*_1_–*N*_4_) at which flank wear was measured. Below the diagram, the angle of rotation/deformation *ϕ* [°]at the pitch point and the calculated average linear flank wear *l* [mm] at certain time sequences of Onyx optimized gear are shown. Table 6 presents quantitative values of rotation angle and average linear flank wear of the Onyx optimized gear observed under torque 1.1 Nm at 4 different cycle sequencies.

**Table 6:** Cumulative quantitative values of rotation angle and average flank wear of the optimized Onyx gear mated with 42CrMo4+QT steel gear under 1.1 Nm at different cycle sequences.

Load cycles, *N* [−]	Rotaion angle, *ϕ* [°]	Average flank wear, *l* [mm]
388.188	6.3	0.068
1.901.747	9.7	0.093
3.377.730	12.1	0.153
4.862.228	14.9	0.186

At the start of the testing (*N*_1_), a significant amount of deformation can be detected due to the geometrical irregularities of teeth flank profiles, which causes additional adaptations of the polymer composite gear. This leads to the transitional phenomena explained by the increasing meshing temperature seen in the first phase of the gear running, as shown in Fig. 8. **The running-in** phase, during which the increasing temperature was detected due to the increasing deformation and consequently, the wear rate (*l*_1_ = 0.068 mm) is increased. After the first iteration (*N*_1_), the contribution of the rotation angle is 37 % and flank wear is 42 % of the total rotation angle and wear. The number of cycles after the first iteration (*N*_1_ = 383,188) represents 7 % of the total gear lifetime of the test. The results suggest that the gear pair's running-in phase significantly impacts the deformation and wear rate.

After the running-in phase, the meshing temperature is approximately stabilized, which means the beginning of the **wear-in** phase. In this phase, deformation and wear approximately linearly increases up to the final phase where gear failure occurs. During the wear-in phase, an increasing influence of fatigue can be emphasized, causing the molecular bond forces to weaken in the polymer, as well as destabilization of the cohesion between fibers and matrix This leads to progressively greater deformation and consequent wear of the tooth due to changing contact conditions between the steel and Onyx gears.

In the last phase/failure phase (after *N*_4_), a significant increase in meshing temperature fluctuation is observed, which is a consequence of the crack progression in the root area of the observed tooth. These phenomena are also confirmed by Fig. 5, where microscopic images of the crack progression in the last part of the optimized Onyx running are shown.

The values of the flank wear rate for the Onyx material have confirmed that the influence of wear must be considered when calculating the fatigue bending strength of the material named as an S–N or Woehler curve. The reason for the formation of abrasive wear is the sliding of the gear flanks (Steel/Onyx) against each other, which leads to the loss of material on the Onyx gear flanks and degradation of the load-bearing properties of the composite gears over an extended period of operation.

The S–N line describes the correlation between bending stress depending on load cycles in the loading fatigue regime. The rule for modeling the S–N line according to VDI2736 [47] is to have three loading points with at least three repetitions at the same failure mode. At the testing, melting occurred at 1.5 Nm, and the tooth root fractured at 1.3 Nm and 1.1 Nm. There were only two relevant data about the obtained number of load cycles with associated gear tooth root failure.

**Fig. 9:**
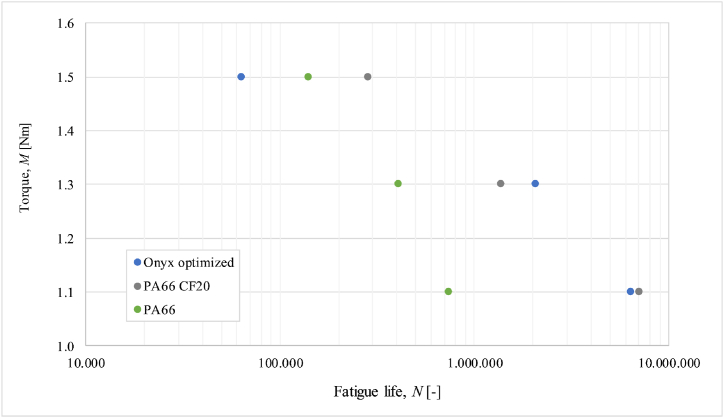
Fatigue life results for optimized Onyx in comparison to conventional machine cut gears.

Fig. 9 illustrates fatigue life expressed in load cycles depending on applied torque on the observed gear pair. In addition, for appropriate evaluation of Onyx durability and comparison purposes,

results for fatigue life of PA66 CF20 (short carbon reinforced polyamide 66) machine-cut gears, and basic PA66 (Polyamide 66) from past research, are also presented in Fig. 9. Both gear materials were paired with 42CrMo4+Qt steel gear, considering the same geometry and loading conditions of gear pair running to have a relevant comparison.

As the number of cycles increased, the fatigue life of Onyx gears is approaching to the machine cut short carbon-reinforced PA66. The lower resistance to fatigue loading of Onyx, compared to the machine-cut gears in Fig. 9, can be attributed to its relatively inferior mechanical properties such as modulus of elasticity, tensile strength, and flexural strength. Additionally, the relatively high deformation of Onyx gear teeth occurred during the meshing process in the running-in phase. Enhancing the toughness of Onyx could contribute to refining the additive manufacturing process to ensure the material flows uniformly along the entire profile of the teeth. The results suggest that carbon fibers in the Polyamide 66 matrix have a positive effect on the prolonged durability of the polymer material, which is a key factor in withstanding permanent fatigue loading during the meshing process.

**Fig. 10:**
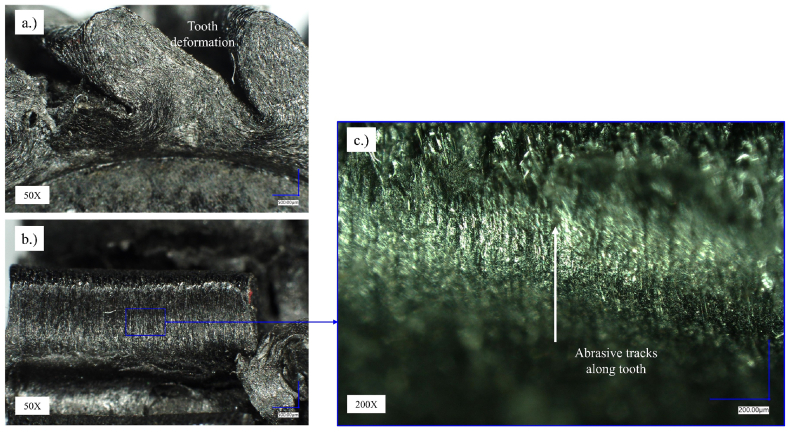
Optical microscopy of the Onyx gears (sample: *M* = 1.5 Nm, *N* = 66.398 load cycles).

**Fig. 11:**
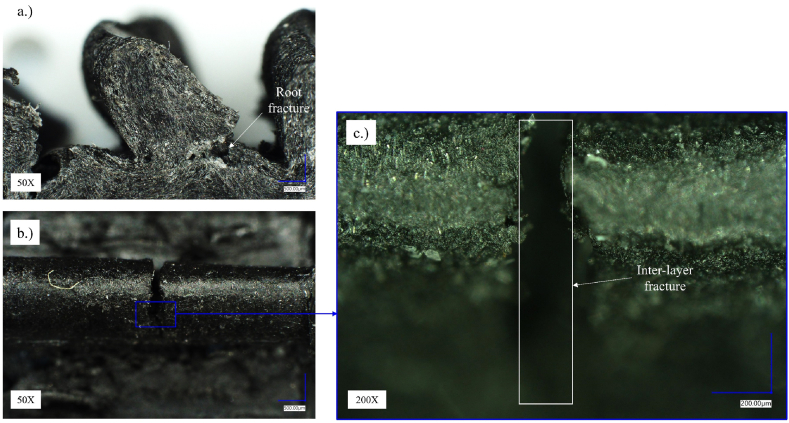
Optical microscopy of the Onyx gears (sample: *M* = 1.3 Nm, *N* = 1.335.334 load cycles).

**Fig. 12:**
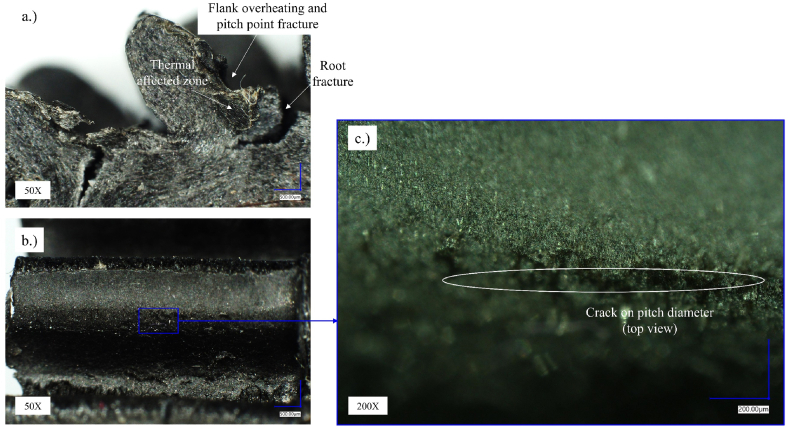
Optical microscopy of the Onyx gears (sample: *M* = 1.1 Nm, *N* = 5.398.691 load cycles).

For a detailed analysis of the wear mechanisms on the Onyx's gear flanks, optical microscopy was conducted using the Keyence VH-7000 series presented in Fig. 10, Fig. 11, Fig. 12 using the same range of magnification for each positioned image in the specific Figure. Fig. 10 presents a gear sample tested under 1.5 Nm. In Fig. 10a, there is a clear presentation of a characteristic gear failure mode called melting which is connected with overheating of the teeth flanks due to excessive bending loads. Fig. 10b presents the top view of the tooth presented in Fig. 10a Fig. 10c presents material degradation on the flank surface of the Onyx gear under magnification 200×. Abrasive tracks along the tooth height occur due to the sliding of the metal gear on the side of the Onyx gear. The deformability of the driven gear made of Onyx material creates the phenomenon of increased sliding, which means, from the point of view of the mechanics of power transmission, changed contact conditions between the gear pair and, as a result, the formation of wear on the less resistant gear, which in this case is the Onyx gear.

Fig. 11 presents the microscopic images of the tested Onyx gear under 1.3 Nm torque. Fig. 10a presents tooth root fracture of the Onyx gear which is the result of long-term bending fatigue caused by driving steel gear. Fig. 11b presents entire the tooth presented from the top view. Fig. 11c shows in more detail the interlayer fracture of the 3D-printed layers in the middle of the gear width due to the effect of contact pressure from the steel gear.

Fig. 12 presents the Onyx gear tested under 1.1 Nm torque. The test sample was fractured in the tooth root area as well and gradual degradation of the flank surface can be detected around pitch diameter where contact pressure is maximal during gear pair meshing (Fig. 12a). The root fracture occurred due to long-term fatigue obtaining over 8 million load cycles. Comparing the state of the tooth flank surfaces in Fig. 10, Fig. 11, Fig. 12b, it can be seen that the surfaces in Fig. 11, Fig. 12 are less damaged than in Fig. 10b, which could be the result of lower contact stresses.

## 4 Conclusions

The paper describes the characterization of a carob reinforced PA66 gears that are additively manufactured, for prototyping purposes. The work includes a comprehensive presentation of the manufacturing process of test gears and testing of the gear pair (steel/Onyx) for the duration until the Onyx gears failure. During operation, the temperature response in the contact between the metal and Onyx gear and the flank wear of the Onyx gear were characterized. Fatigue life data of the Onyx gear are presented and compared to conventionally produced gears from the PA66-based composite and virgin material. Based on the given results, the following conclusions can be obtained:

- The characteristic running of the 42CrMo4+QT/Onyx gear pair has been detected by the measured average surface meshing temperature response in contact between gear pairs, which is similar to the running of conventionally produced polymer-based gears mated with steel pinion.
- It has been found two typical failure modes of Onyx gears which are melting and failure in tooth root due to fatigue. Melting occurs at the highest load (torque 1.5 Nm) due to overheating tooth flank what is a consequence of overloading conditions. Fatigue is characteristic which appears at lower torques where the gear pair is exposed to fatigue.
- During gear pair running, significant deformation of the Onyx tooth is detected and proved by expressed measured rotation angle (in the final phase: 14.9°) of the tooth at the pitch point where flank pressure caused by steel gear is the highest. It has been found that deformation of the tooth increases progressively during an entire lifetime. Relatively high deformation of Onyx teeth can occur due to a relatively low modulus of elasticity compared to traditional machine cut gears.
- Measurements of flank wear at certain gear diameters were performed in a certain number of reached load cycles. It was proved that wear progressively increases during gear running. The maximum average flank wear of Onyx gear was 0.186 mm which is in line with expectations compared to machine cut gear (PA66 CF20) (*l* = 0.1082 mm), considering the same geometry obtained cited in the paper Hriberšek and Kulovec [48].
- Comparing the failure life of Onyx gear to machine cut PA66 CF20, Onyx has promising failure life, especially at lower bending stress where failure life compared to machine-cut gearis approximately the same. It gives material important value for using it in new prototyping concepts for gear design.

The guidelines for future research work will be to enhance the longer durability of the Onyx gear exposed at higher loads, specifically at 1.5 Nm. A potential activity that could ensure the longer lifetime of the Onyx gear is modifications to the design of the 42CrMo4+QT steel gear in tip relief, which would result in lower bending stresses in the root area of the Onyx gear, and consequently lead to a longer lifespan of the composite gear. To accomplish the mentioned activity, the tool design should be modified first and then manufactured.

## Data availability statement

Data will be made available on request.

## CRediT authorship contribution statement

**Matija Hriberšek:** Writing – review & editing, Writing – original draft, Methodology, Investigation, Formal analysis, Data curation, Conceptualization. **Simon Kulovec:** Resources, Funding acquisition. **Awais Ikram:** Writing – original draft, Methodology, Investigation. **Matjaž Kern:** Methodology, Formal analysis, Conceptualization. **Luka Kastelic:** Investigation, Conceptualization. **Franci Pušavec:** Writing – review & editing, Resources, Funding acquisition, Conceptualization.

## Declaration of competing interest

The authors declare that they have no known competing financial interests or personal relationships that could have appeared to influence the work reported in this paper.
